# Circumferential Strain Can Be Used to Detect Lipopolysaccharide-Induced Myocardial Dysfunction and Predict the Mortality of Severe Sepsis in Mice

**DOI:** 10.1371/journal.pone.0155346

**Published:** 2016-05-13

**Authors:** Ming Chu, Yao Gao, Bin Zhou, Bingruo Wu, Junhong Wang, Di Xu

**Affiliations:** 1 Department of Geriatrics, First Affiliated Hospital with Nanjing Medical University, Nanjing, China; 2 Departments of Genetics, Pediatrics and Medicine (Cardiology), Albert Einstein College of Medicine of Yeshiva University, New York, United States of America; 3 Department of Cardiology, First Affiliated Hospital with Nanjing Medical University, Nanjing, China; University of Leicester, UNITED KINGDOM

## Abstract

**Background:**

Sepsis-induced myocardial dysfunction is a common and severe complication of septic shock. However, conventional echocardiography often fails to reveal myocardial depression in severe sepsis. Recently, strain measurements based on speckle tracking echocardiography (STE) have been used to evaluate cardiac function.

**Aims:**

To investigate the role of STE in detecting lipopolysaccharide (LPS)-induced cardiac dysfunction, M-mode and 2-D echocardiography were used in LPS-treated mice.

**Methods:**

The mice were treated with a 10mg/kg (n = 10), 20mg/kg (n = 10) or 25mg/kg LPS (n = 30) to induce cardiac dysfunction. Subsequently, the ejection fraction (EF) and fractional shortening (FS) were measured with standard M-mode tracings, whereas the circumferential (Scirc) and radial strain (Srad) were measured with STE. Serum biochemical and cardiac histopathological examinations were performed to assess sepsis-induced myocardial injury.

**Results:**

20mg/kg LPS resulted in more deterioration, myocardial damage and cardiac contractile dysfunction based on serum biochemical and histological examinations. The mice that were subjected to 20mg/kg LPS exhibited reduced Scirc but no reduction in Srad, whereas on conventional echocardiography, the ejection fraction (EF) and fractional shortening (FS) were similar in the 10mg/kg and 20mg/kg groups. Moreover, Scirc was positively correlated with body temperature in the mice at 20 h after LPS injection (r = 0.746, p = 0.001), but no significant correlation was observed between Srad and body temperature (r = 0.356, p = 0.123). Moreover, the mice with high Scirc (-5.9% to -10.4%) exhibited reduced mortality following the administration of 25mg/kg LPS (p = 0.03) compared with the low-strain group (-2% to -5.9%).

**Conclusions:**

Taken together, our findings indicate that circumferential strain is a specific and reliable indicator for evaluating LPS-induced cardiac dysfunction in mice.

## Introduction

Sepsis is responsible for millions of deaths worldwide each year and is a frequent cause of death among people who have been hospitalized. Septic cardiomyopathy is a well-described complication of severe sepsis and septic shock[1]. Septic cardiomyopathy contributes to multi-organ failure due to insufficient vascular perfusion pressure[2]. Although increasing evidence suggests that enhanced production of a large number of inflammatory cytokines can directly or indirectly cause cardiac dysfunction, the precise mechanisms for myocardial dysfunction in sepsis remain undefined[1,3,4]. There are controversies regarding the pathophysiology of sepsis-induced myocardial depression and its treatment strategies, many of which are still in the experimental phase. Experimentally, the administration of lipopolysaccharide (LPS) to laboratory animals, especially mice, has been widely used to study the mechanisms of septic cardiomyopathy[5].

Echocardiography is an excellent noninvasive tool for the assessment of ventricular size and both systolic and diastolic function, and it is routinely used in patients with heart failure[6]. Conventional echocardiogram-derived left ventricular ejection fraction (LVEF) measures may be suitable for diagnosing sepsis-induced myocardial dysfunction based on a reduction in the afterload[7]. Additionally, the LVEF may be particularly misleading among non-survivors because the LVEF may remain normal or near normal despite compromised stroke volume due to the failure of the LV to dilate and hence boost stroke volume via the Frank-Starling mechanism[8]. Thus, in one of the most common causes of heart failure, the ejection fraction does not characterize the extent of disease. Ejection fraction is also of limited use for early forms of other cardiomyopathies that are characterized by regional, rather than by global myocardial dysfunction[9]. Recently, a novel echocardiographic imaging technique, i.e., speckle tracking echocardiography (STE), which is based on myocardial strain analysis, has been demonstrated to a clinically useful tool for quantifying cardiac function[10,11]. In a variety of experimental and clinical settings in asymptomatic conditions, STE can detect left ventricular dysfunction by serially capturing segmental tissue motion in multiple planes and axes over the cardiac cycle that cannot not be detected using conventional systolic echocardiographic measures of left ventricular function[9,11,12]. Recent studies have demonstrated that left ventricular strain and torsion are impaired in septic patients as assessed with STE[13,14].

In the present study, we hypothesized that circumferential strain is a specific and reliable indicator for the evaluation of dose-dependent-LPS-induced cardiac dysfunction in mice and thus may be beneficial for the diagnosis and prognostication of septic cardiomyopathy.

## Materials and Methods

### Animals

All procedures involving the mortality-related aspects of the protocol described in this study were approved by the Nanjing Medical University Committee on Animal Care. All experiments were performed according to the guidelines of the Principles of Laboratory Animal Care and the Guide for the Care and Use of Laboratory Animals published by the National Institutes of Health. All efforts were made to minimize suffering.

The experiments were performed on 5-10-week-old male C57BL/6J mice weighing 23–26 g. The animals were obtained from the Nanjing University Model Animal Research Center. The animals were housed 6 per cage under standard laboratory conditions for at least 10 days on a 12 h/12 h light/dark cycle at 21–23°C. All animals received standard laboratory diet and water ad libitum. During the experiments, the body temperature was rectally determined using a THM 150 temperature probe (Indus Instruments, Houston, TX, USA) at a depth of 1.5 cm on the conscious mouse. The mice were quickly anesthetized with 1.0% pentobarbital sodium, and blood was immediately collected from the retro-orbital sinus. The blood was allowed to clot at room temperature for 10 min, and the serum was obtained by centrifugation (approximately 1200× g for 10 min at 4°C) and stored at −80°C until use.

### Septic cardiomyopathy model

Lipopolysaccharide (LPS) administration was used to induce sepsis and septic cardiomyopathy as previously described[15]. E. coli LPS (serotype O111:B4, Sigma-Aldrich, St Louis, MO, USA) was dissolved in sterile physiological saline (0.9% NaCl) at a concentration of 1 mg/ml. The mice were intraperitoneally injected with 10, 20 or 25 mg of LPS per kg body weight. At the end of experiments, after conventional echocardiographic and speckle tracking echocardiographic (STE) measurements and blood collection were performed, the hearts were removed under isoflurane anesthesia and fixed in 10% phosphate-buffered formaldehyde for histology analyses.

### Experimental protocol

Two-dimensional echocardiographic LV short-axis images were assessed. The FS and EF were measured from conventional M-mode tracings, whereas the Scirc and Srad were derived offline by STE using Echopac PC software (Version 12.0.0, GE Vingmed).

To distinguish the different degrees of cardiac dysfunction in severe sepsis, 20 C57BL/6J mice were divided into two groups according to the dose of LPS administered; ten mice were injected with 10 mg/kg LPS, and the others were treated with 20 mg/kg LPS. Six hours after injection, conventional M-mode echocardiography parameters were assessed. Additionally, STE was used to obtain the Scirc and Srad values. We used humane endpoints and euthanized the animals prior to the end of the experiments. Weight loss, depression, inappetence, extreme reluctance to stand coupled with difficulty in breathing, low body temperature, weakness and moribund status, seizures, severe diarrhea, and paralysis of one or more extremities were the signs we used to determine the time at which the animals should be euthanized. Anesthesia (sodium pentobarbital) was used to reduce the suffering and distress of the animals before any procedure that was potentially painful or stressful. We observed and monitored the health of the animals every hour, and there were no unexpected deaths among these animals.

Subsequently, thirty C57BL/6J mice injected with 25 mg/kg LPS were assessed to determine the relationship between circumferential strain and survival rate. Six hours following injection with LPS, short-axis 2D echocardiography data were acquired, and the Scirc values derived from STE was assessed. For this experiment, mortality was assessed every 3 hours for 5 days. When a mouse was judged too ill to survive until the next time point, it will be sacrificed (anesthetized with sodium pentobarbital) and considered dead due to sepsis for humane reasons in order to reduce the suffering and distress of the animals.

### Conventional Echocardiography

Echocardiography was conducted while the mice were anesthetized with 1.0% isoflurane under light sedation at a room temperature of 22°C and with decreased ambient lighting, while they were maintained in a supine left decubitus position by an experienced handler. Using a Vivid 7 ultrasound machine (Vivid7, GE Medical Systems, Milwaukee, Wisconsin) equipped an il3L linear probe operated at 14 MHz, the hearts were imaged in M-mode at the mid-papillary level in the parasternal short-axis view and in 2-D mode in the parasternal long- and short-axis views. All mice underwent at least one echocardiography measurement for acclimation to the procedure. Echocardiographic measurements were obtained from grayscale M-mode images at the mid-papillary level in the parasternal short-axis view and in 2-D mode in the parasternal long- and short-axis views. The conventional measurements of the LV included the following: left ventricular internal diameter at diastole (LVIDd), left ventricular internal diameter at systole (LVIDs), left ventricular volume at diastole (LVVd), left ventricular volume at systole (LVVs), ejection fraction (EF), and fractional shortening (FS).

### Speckle Tracking Echocardiography

Each of two coplanar, orthogonal linear probes acquired forty-one frames from one heart cycle. During each cardiac cycle, the left ventricle undergoes a complex functional pattern of tissue deformation in multiple planes. In the circumferential axis, myocardial shortening during systole followed by the opposite changes during diastole can be observed as myocardial deformations. Based on the Lagrangian and Eulerian strain tensors of finite deformation theory, the extensional strain of the soft tissue in a pre-specified direction can be defined as the change in the length of a segment divided by its original length ([L1−L0]/L0)[16]. Speckle tracking-based strain analyses of the myocardial motion (in the short-axis images) integrates frame-to-frame data from cine loops, which allows for measurements of segmental and global myocardial strains in the circumferential axes. These measures are plotted as curvilinear data for each region tracked. Strain is a negative value; the more negative the value, the greater the degree of deformation and the better the function. The speckle-tracking based strain analyses were performed offline. All images were analyzed three times to ensure the accuracy of the results. The data were analyzed by two independent investigators. The cardiac STE measurements included 2D speckle tracking of circumferential and radial strain.

### Biochemical Analysis

We used the Roche CARDIAC Troponin T Quantitative test from Roche Diagnostics GmbH (Mannheim, Germany) to determine serum cardiac troponin-T (cTnT). Moreover, we used a VITROS 5600 automated biochemical analyzer (Ortho-Clinical Diagnostics, New York, USA) to test the serum CK-MB, ALT and AST levels. The samples were analyzed at the central laboratory of Nanjing Medical University First Affiliated Hospital.

### Histopathological Examination

At the end of experiment, the hearts were removed under isoflurane anesthesia after conventional echocardiographic and STE measurements and blood collections and cut into transverse blocks (2 mm thick) at the level of the papillary muscles. Next, the hearts were immersion-fixed in 4% buffered paraformaldehyde and embedded in paraffin for histological analysis. Serial sections of 4 μm were cut and subjected to hematoxylin and eosin staining (H&E). The histological changes in erythrocyte leakage and leucocyte infiltration into the cardiac interstitium were examined under a light-microscope. The myocardial leucocytes were counted according to the previously described methods[17]. The analysis of the histologic samples was blinded, i.e., the person who analyzed the histologic samples was blinded to the treatment. The infiltration of myocardial leucocytes is expressed as the average number of leucocytes per field (n = 8-10/group).

### Statistical Analysis

The mean ± SEM was determined for each study group. Comparisons between the groups were performed using one-way ANOVA tests. The relationships between the strain parameters and the body temperatures of the mice following LPS administration were examined with simple linear regression analyses and correlation analyses. The survival curves were compared with the log-rank test. All statistical tests were two-sided, and discrepancies with p<0.05 were considered statistically significant. All statistical analyses were performed using SPSS software (Version 16.0, SPSS Inc., Chicago, Illinois).

## Results

### Different degrees of LPS-induced cardiac dysfunction assessed according to myocardial performance

**Fig 1:**
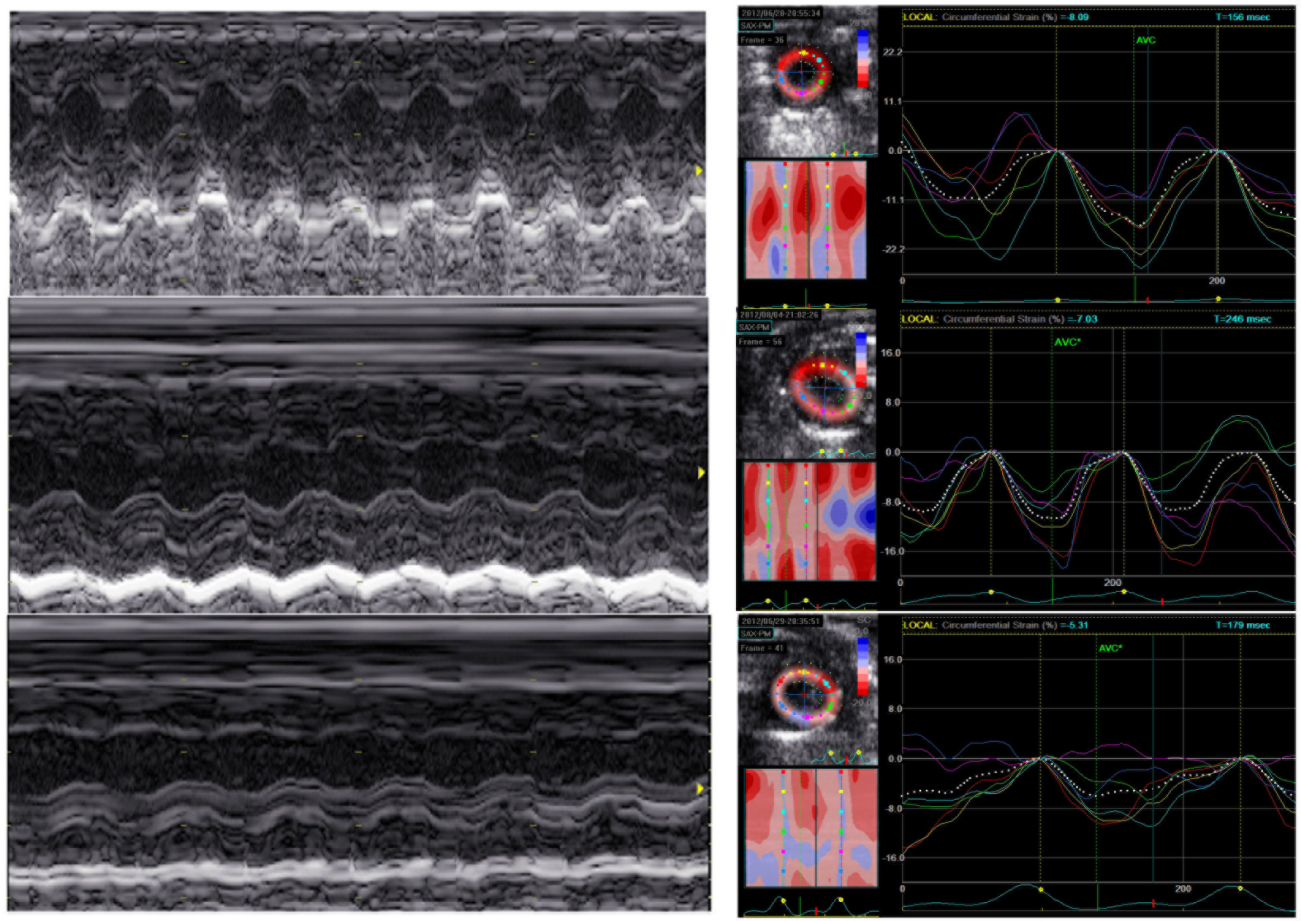
Examples of left ventricular myocardial function assessments by conventional echocardiography (left) and strain echocardiography (right) at baseline (upper plane), after 10 mg/kg LPS challenge (middle plane), and 20 mg/kg LPS administration (lower plane), respectively.

**Fig 2:**
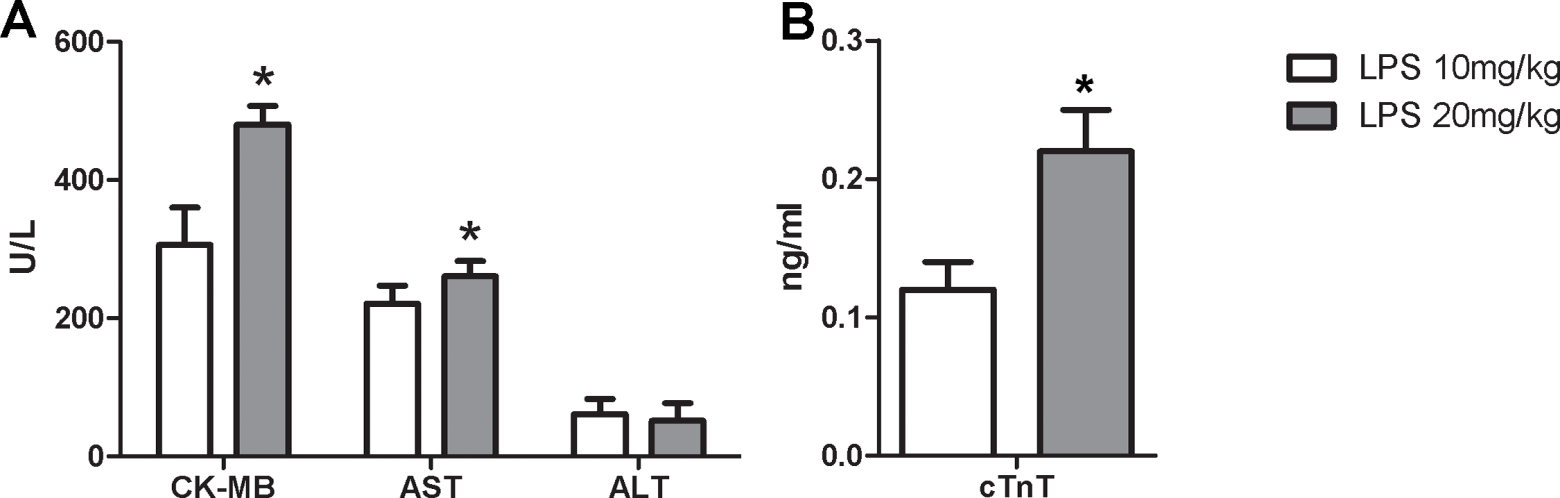
Serum biochemical examination showed different degrees of myocardial damage. *p<0.05, vs. LPS 10mg/kg group, n = 10 per group.

**Fig 3:**
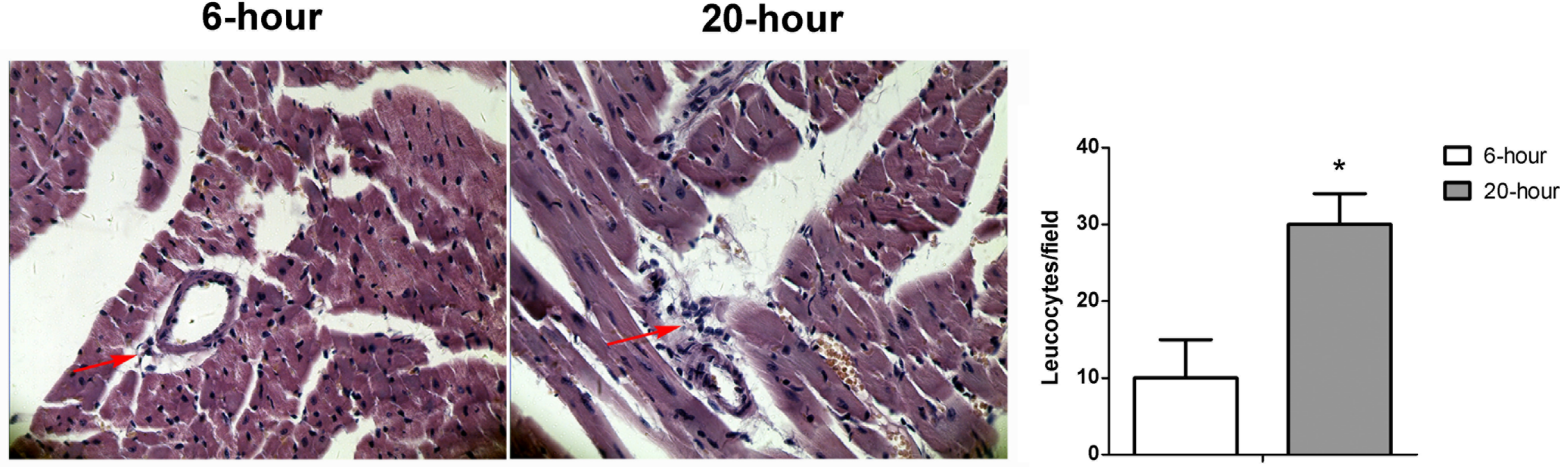
LPS-induced leukocyte infiltration into interstitial myocardium was increased after 20mg/kg LPS administration (left) relative to 10mg/kg group (middle). Cardiac tissues at the papillary muscles level were harvested 6h after LPS treatment. Paraffin sections were prepared and stained with HE. The histological changes of leukocyte infiltration were detected under a light-microscope. 200 ×. * p<0.05, vs. LPS 10mg/kg group, n = 10 per group.

To explore whether strain imaging was able to distinguish different degrees of LPS-induced cardiac dysfunction, two groups of mice were treated with low or high doses of LPS. Interestingly, conventional echocardiography revealed no difference in global cardiac function between the 10mg/kg and 20mg/kg groups (Fig 1). Moreover, both the 10mg/kg and 20mg/kg LPS-injection groups exhibited an increase in myocardial volume and decreases in EF and FS, whereas no significant differences in these parameters were observed between the two LPS-treated groups (Table 1). Global circumferential strain analysis revealed significant deteriorations of LV myocardial function between the start of the experiment and 6 hours after both low- and high-dose LPS challenge (Table 1). The sLV strain decreased in all animals during sepsis, but the 20mg/kg group exhibited a significantly greater decrease in circumferential strain than the 10mg/kg group (Table 1). To further prove the different degrees of LPS-induced cardiac dysfunction, serum biomarker and leukocyte infiltration analyses were performed. As expected, high-dose LPS administration resulted in higher levels of serum cTnT (Fig 2B), CK-MB, ALT, and AST (Fig 2A) and increased leukocyte infiltration (Fig 3) compared with the 10mg/kg group.

**Table 1:** Cardiac Function Measured by Conventional and Strain Echocardiography.

	LPS 10mg/kg(n = 10)		LPS 20mg/kg(n = 10)	
Variables	baseline	6-hour	baseline	6-hour
LVIDd(mm)	3.11±0.23	3.23±0.32	3.02±0.25	3.96±0.50 *
LVIDs(mm)	1.87±0.19	2.38±0.37 *	1.81±0.23	2.52±0.35 *
LVVd (μl)	77.50±17.53	88.20±25.23	78.80±14.74	97.50±40.27
LVVs (μl)	17.50±4.63	38.20±15.37 *	18.70±4.31	43.80±18.47 *
EF(%)	77.25±2.96	58.45±7.94 *	79.15±2.66	54.75±4.77 *
FS(%)	40.00±2.83	26.55±4.78 *	41.50±3.03	23.88±2.70 *
Srad(%)	27.55±10.67	13.28±8.49	28.95±9.55	17.23±6.83
Scirc(%)	-14.65±3.00	-8.48±1.72 *	-15.15±2.73	-5.74±2.52 *

Data are given as mean ± standard deviation. LPS, Lipopolysaccharide; LVIDd, left ventricular internal diameter at diastole; LVIDs, left ventricular internal diameter at systole; LVVd, left ventricular volume at diastole; LVVs, left ventricular volume at systole; EF, ejection fraction; FS, fractional shortening; Scirc, circumferential strain; Srad, radial strain.

*p<0.05, vs. baseline group.

### Correlation of circumferential strain with hypothermia

**Fig 4:**
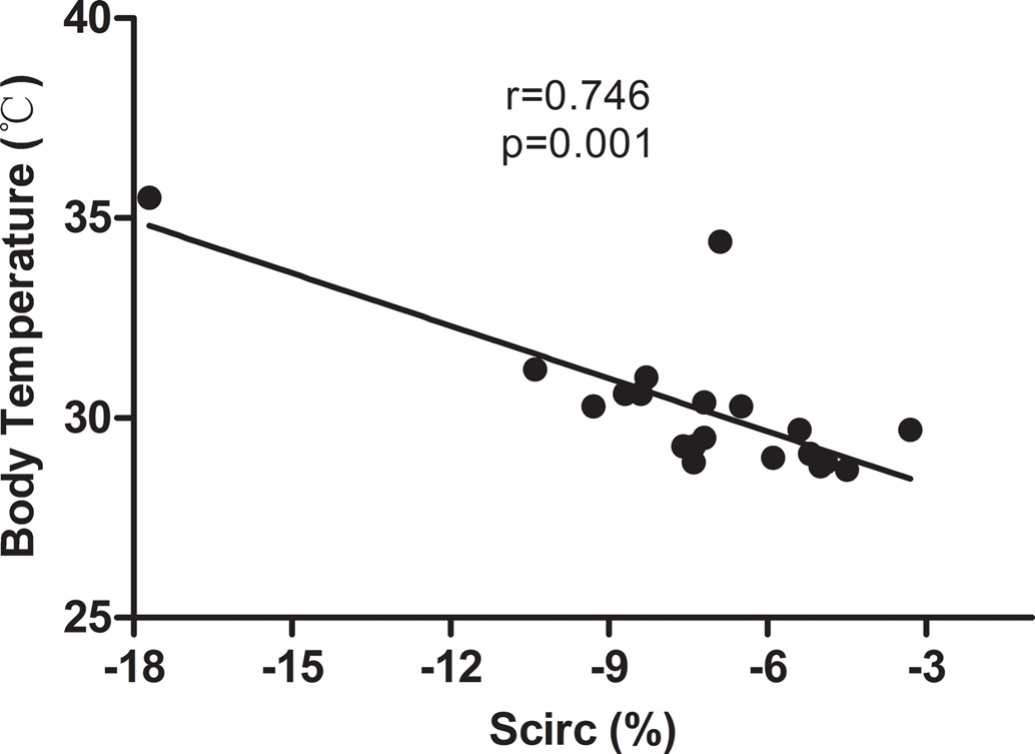
Correlations between circumferential strain, estimated by speckle tracking echocardiography, and body temperature of mouse models following LPS-induced sepsis.

Numerous lines of evidence have revealed that the severity of hypothermia during the early phase of sepsis or endotoxemia can predict the mortality of individual animals[18,19]. We determined the body temperatures of the mice 20 hours after LPS treatment. As illustrated in Fig 4, a positive correlation was observed between Scirc and body temperature (r = 0.746, p = 0.001). However, no statistical correlation was noted between Srad and body temperature (r = 0.356, p = 0.123).

### Circumferential strain as a predictor of mortality

**Fig 5:**
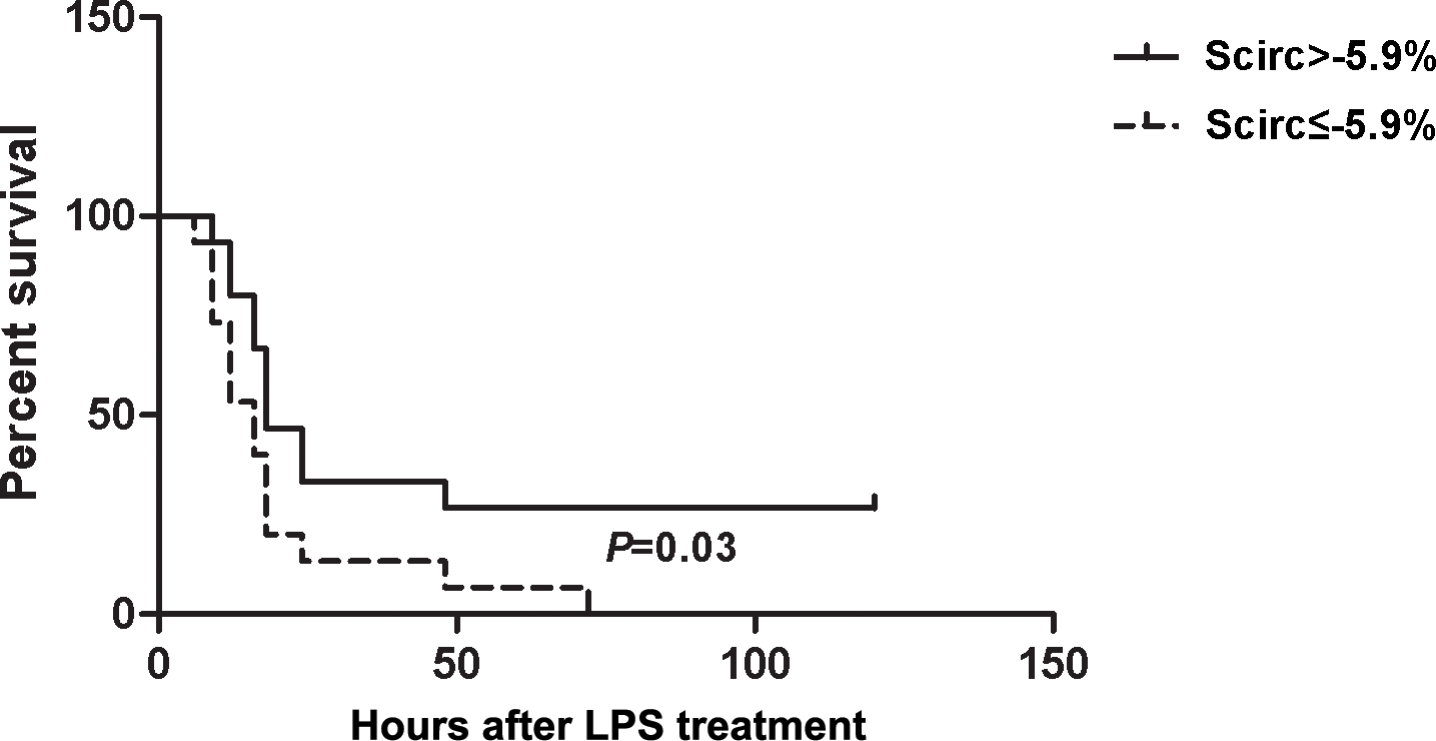
Survival curves of all mice divided into two groups according to their median value of circumferential strain (Scirc): ≤-5.9% and >-5.9%. The animals were monitored every 3 hours for up to 120 hours following LPS injection (25mg/kg). n = 15 per group.

The left ventricular circumferential strain measurements were assessed 6 h after LPS injection. The median circumferential strain value of all mice was -5.9% as derived from STE. All mice were subsequently divided into 2 groups according to the median value of all of the Scirc measurements as follows: ≤-5.9% (-2% to -5.9%, n = 15), and >-5.9% (-5.9% to -10.4%, n = 15). At the end of our experiment, totally 26 mice died in 25mg/kg LPS-treated group. Of which, 3 were sacrificed for humane reason after they were judged to be too ill to survive until the next time point. Furthermore, the high-strain group (>-5.9%) displayed a significantly higher survival rate than the low-circumferential-strain group (p = 0.03) (Fig 5).

## Discussion

In the present study, we investigated the roles of circumferential strain in the detection of LPS-induced myocardial dysfunction and the prediction of mortality due to severe sepsis in a mouse model. Our study demonstrated that strain echocardiography can be used to distinguish different degrees of LPS-induced cardiac dysfunction. Furthermore, we found that circumferential strain was positively correlated with body temperature following LPS administration in the mouse model and predicted mortality due to septic cardiomyopathy.

Although myocardial dysfunction is common in severe sepsis, its underlying physiopathology is not completely understood. Most currently used animal sepsis models have demonstrated that septic shock is associated with lethally reduced cardiac output and elevated systemic vascular resistance[20,21]. The prognostic value of myocardial depression in sepsis is controversial. Recent studies[22] have related the risk of fatal outcomes from septic shock to the intensity of myocardial depression, but the Francois group found that LVEF as assessed with conventional echocardiography does not predict the outcomes of individual patients[23]. Several studies have demonstrated that there are significantly altered loading conditions in mouse models of LPS-induced cardiac dysfunction[24–26]. Our results revealed that LPS administration resulted in hypothermia and decreased heart rate that were indicative of significant hemodynamic changes in LPS-induced septic shock. Altogether, significant hemodynamic changes reduce the accuracy of conventional echocardiography in cases of severe sepsis. In this study, we found that both the low- and high-dose LPS-injection groups exhibited increased myocardial volumes and decreased EF and FS, whereas significant differences in these parameters were not observed between the two LPS-treated groups. However, the serum biomarker and leukocyte infiltration analyses revealed the high-dose LPS administration resulted in higher levels of serum cTnT, CK-MB, ALT, and AST and increased leukocyte infiltration compared with low-dose LPS, and these findings were consistent with the STE measurements. However, inhaled isoflurane has been demonstrated to reduce afterload and left ventricular function[27]; the use of this anesthetic represents one of the limitations of this study, and this issue requires further study.

Mouse cardiac muscles contain fibers that extend in three different directions, i.e., longitudinally, helically, and in rings. STE recognizes myocardial deformation in different directions and can therefore not only distinguish longitudinal motion but can also distinguish circumferential and radial motion[28]. In the present study, we found that decreased circumferential strain was useful for distinguishing different degrees of sepsis-induced cardiac dysfunction, whereas the load-dependent indexes, such as EF and FS, were not useful for distinguishing this difference. Bauer et al reported that compared to conventional echocardiographic measures of left ventricular function, the peak longitudinal strain and strain rate were better able to detect changes in adult mouse hearts at earlier time points following myocardial infarction and to predict the later development of adverse LV remodeling[22].Indeed, echocardiographic strain-based measures provide more sensitive and rapid assessments of cardiac function in mice following myocardial infarction and in response to cardiac therapy. Recent studies have reported that torsion and strain abnormalities identified by strain imaging are present in septic patients without major impairments of left ventricular ejection fraction[13,14]. Therefore, the more sensitive indexes from speckle tracking imaging demonstrated an obvious advantage in the evaluation of myocardial depression during severe sepsis compared with conventional echocardiographic indexes. However, STE is a novel technology that has numerous analysis indexes and includes multiple motion indicators. Many indicators from strain echocardiography were not considered in the present study due to the small sizes of the hearts of mice. Further work is needed to establish a standard evaluation system based on STE for the evaluation of septic cardiac dysfunction.

Another interesting discovery in our study is that the Scirc was positively correlated with body temperature and predicted the mortality of mice with severe sepsis. Previous studies have demonstrated that regional midwall strain is influenced by both global remodeling and regional fibrosis after myocardial infarction[29]. Another study revealed that radial and circumferential strain distinguishes infarcted from viable myocardia[30]. Therefore, strain measurements from STE can be used independently to detect myocardial damage as corroborated by our results. Temperature instability over a range from hyperthermia to hypothermia is one of the hallmarks of sepsis because definitions of sepsis include temperatures higher than 38°C and below 36°C[31]. An early study demonstrated a strong correlation between the serum level of IL-6 and the degree of hypothermia in sepsis[19]. Moreover, in our study, the positive correlation between Scirc value and body temperature could be an indication of consistent changes in local myocardial damage and systemic circulatory failure during septic shock. Satio et al also demonstrated that the severity of hypothermia is closely related to mortality in mice with septic shock[19]. Taken together, these results indicate that LPS challenge results in decreased circumferential strain, which is an indicator of myocardial damage and contributes to hypothermia and death.

Our study has several clinical implications. Myocardial contractile failure is an important manifestation of severe sepsis and septic shock and is associated with a poor prognosis. Echocardiography plays an indispensable role in cardiac imaging due to its real-time acquisition and ease of maneuverability. However, novel echocardiographic approaches, such as speckle tracking imaging (STI), are continuously being developed. Thus, the question of whether it is necessary to measure large numbers of indicators via combined conventional echocardiography and STE emerges. Additionally, if the indexes from different system are divergent, which is the more reliable predictor of the physiological process, especially when patients have developed symptoms of suspected heart failure? Our study indicates that it is necessary to combine conventional echocardiography with STE in cases of severe sepsis and septic shock, although circumferential strain was more sensitive and reliable in evaluating cardiac inotropism in unstable hemodynamic states compared with EF and FS. Furthermore, circumferential strain has the prognostic value of being able to predict mortality. However, this mouse model is quite resistant to endotoxins and exhibits high heart rates, distinct hemodynamic changes, and limited blood volumes compared with humans[32]. Future work is needed to determine standard cutoff values for circumferential strain that are useful in terms of the prognoses of humans with septic cardiomyopathy.

In conclusion, this study suggests that strain echocardiography can be used to distinguish different degrees of LPS-induced cardiac dysfunction and predict the mortality of severe sepsis in mice. We propose that STE can be used to improve the diagnostic and prognostic roles of sepsis-induced myocardial depression in mice. Certainly, further clinical studies are needed to improve the clinical diagnosis and treatment of cardiac dysfunction.
